# Double‐Filtration Plasmapheresis Versus Efgartigimod for Generalized Myasthenia Gravis: Severity‐Stratified Benefits in a Prospective Observational Multicenter Study

**DOI:** 10.1002/cns.70838

**Published:** 2026-03-16

**Authors:** Kan Wang, Hanyu Xin, Mengze Zhang, Qiuju Li, Yanan Wu, Xia Li, Jing Peng, Chong Xie, Xiajun Zhou, Li Gao, Xuzhong Pei, Yiwei Yang, Yuhui Wang, Yuan Zhong, Lu Zhang, Desheng Zhu, Yangtai Guan

**Affiliations:** ^1^ Department of Neurology, Renji Hospital, School of Medicine Shanghai Jiao Tong University Shanghai China; ^2^ Department of Neurology Punan Branch of Renji Hospital, School of Medicine, Shanghai Jiao Tong University (Punan Hospital in Pudong New District, Shanghai) Shanghai China; ^3^ Department of Neurology Anhui No. 2 Provincial People's Hospital Hefei China; ^4^ Hangzhou Dian Medical Laboratory Co., Ltd. Hangzhou China; ^5^ Zhejiang Key Laboratory of Digital Technology in Medical Diagnostics Hangzhou China; ^6^ School of Medicine Shanghai Jiao Tong University Shanghai China

**Keywords:** clinical efficacy, double‐filtration plasmapheresis, efgartigimod, generalized myasthenia gravis, stratified therapy

## Abstract

**Introduction:**

Double‐Filtration plasmapheresis (DFPP) and efgartigimod (EFG) are both effective acute exacerbation therapies for generalized myasthenia gravis (gMG), yet real‐world comparative evidence remains limited. This study aims to systematically compare their clinical efficacy and safety to guide individualized treatment strategies.

**Methods:**

This prospective multicenter study (Aug 2019–Mar 2025) evaluated gMG patients treated with a cycle of DFPP (3–5 sessions) or EFG (4 weekly infusions). Primary endpoints included changes in Quantitative Myasthenia Gravis (QMG) and Myasthenia Gravis Activities of Daily Living (MG‐ADL) scores from baseline (T0) to post‐treatment (T1) and 1 month after T1 (T2). Secondary endpoints covered clinical meaningful improvement (CMI), deep improvement rates (defined as ≥ 5‐point reduction in MG‐ADL or ≥ 9‐point in QMG), immunological changes, and safety.

**Results:**

Among 66 MG patients (EFG = 41, DFPP = 25), both DFPP and EFG significantly reduced QMG and MG‐ADL scores at T1 and T2. In moderate‐to‐severe gMG (MGFA ≥ IIb), 3/5‐session DFPP was superior to 4‐infusion EFG at T2 (*p* < 0.05), while efficacy was comparable in mild cases (MGFA ≤ IIa). Sustained CMI rates (through T2) increased with higher treatment intensity; the 5‐session DFPP rate was significantly higher than the 3‐session DFPP (85% vs. 45%, *p* = 0.049) and numerically higher than the 4‐infusion EFG (85% vs. 70%, *p* = 0.413). The proportion of patients achieving deep improvement in 5‐session DFPP was higher than 4‐infusion EFG. DFPP broadly reduced immunoglobulins/complement versus EFG's selective immunoglobulin G (IgG) reduction. Adverse events occurred in 28.0% of the DFPP group (mainly catheter‐related thrombosis) and 39.0% of the EFG group (predominantly headache; *p* = 0.431), with no serious events reported.

**Conclusion:**

Both DFPP and EFG effectively treat gMG, though DFPP offers greater benefit in moderate‐to‐severe cases, underscoring the need for individualized therapy.

## Graphical abstract

Both Double‐Filtration Plasmapheresis (DFPP) and efgartigimod effectively treat acute exacerbations of generalized myasthenia gravis (gMG), with efficacy rising across successive sessions. In moderate‐to‐severe gMG, 3/5‐session DFPP outperformed 4‐infusion efgartigimod, while efficacy was comparable in mild cases. DFPP broadly reduced immunoglobulins, complement, and cytokines, whereas efgartigimod selectively lowered IgG, supporting severity‐stratified personalized therapy.

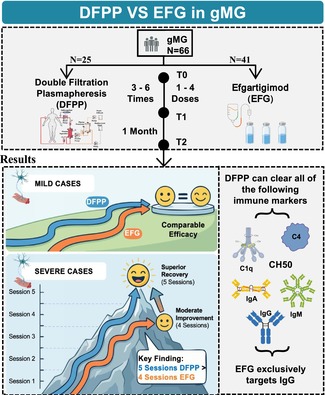

## 1 Introduction

Myasthenia gravis (MG) is an autoimmune disorder mediated by pathogenic antibodies targeting the neuromuscular junction (NMJ). Central to its pathology is the impairment of synaptic transmission by IgG autoantibodies, such as those against the acetylcholine receptor (AChR), leading to recurrent episodes of fluctuating muscle weakness [1]. Globally, MG has a prevalence of approximately 150–250 cases per million people [2, 3]. Although relatively common among rare diseases, it exhibits considerable heterogeneity in both disease severity and clinical phenotype. Current treatment strategies focus on immunomodulation, including non‐specific immunosuppressive agents—such as corticosteroids and conventional immunosuppressants—as well as rapidly acting targeted therapies.

Double‐Filtration Plasmapheresis (DFPP), a conventional first‐line intervention for acute exacerbations, mediates multi‐targeted removal of circulating autoantibodies, complement factors, and cytokines (such as IL‐6, IL‐12), leading to rapid clinical improvement in severe cases [4]. Extensive clinical experience and studies have revealed variable responses to DFPP among MG subpopulations. In patients with muscle‐specific kinase (MuSK) antibody‐positive MG, response rates range from 50% to 93%, significantly higher than those achieved with intravenous immunoglobulin (IVIg; 11%–61%) [5]. Moreover, DFPP has been shown to effectively improve muscle strength during acute worsening or in the perioperative setting for patients scheduled for thymectomy, thereby reducing the risk of postoperative crisis [6]. However, DFPP has notable limitations: it is invasive, requires vascular access, and may cause hypotension, catheter‐related infections, and electrolyte disturbances. Its clinical effect is transient, typically lasting only 1–2 months, and its use is constrained by the need for specialized equipment and a trained medical team, limiting both accessibility and long‐term tolerability.

In recent years, Fc receptor (FcRn) antagonists, represented by efgartigimod (EFG), have been gradually introduced into clinical practice, offering a new direction for the targeted treatment of generalized myasthenia gravis (gMG). By specifically blocking FcRn‐mediated IgG recycling and promoting the degradation of pathogenic IgG, this class of drugs achieves more selective immunomodulation without affecting other immunoglobulins such as IgM or IgA [7]. The pivotal phase III ADAPT trial demonstrated that among AChR antibody‐positive gMG patients, approximately 68% of those receiving EFG met the MG‐Activities of Daily Living (MG‐ADL) responder criteria within the first treatment cycle, significantly higher than the placebo group (30%). EFG also exhibited a favorable safety profile, with the most common adverse events being mild‐to‐moderate headache and upper respiratory tract infections, providing robust evidence for its clinical use [8].

Nevertheless, there is still a lack of research data directly comparing the efficacy and safety of DFPP and EFG in real‐world clinical settings. Previous research has predominantly focused on comparing DFPP with IVIg or EFG with IVIg in patients with AChRAb positive gMG experiencing myasthenic crisis (MC) or impending myasthenic crisis (IMC). Multiple studies have indicated that both DFPP and EFG are superior to IVIg in terms of speed of onset and degree of clinical improvement [4, 6, 9]. However, no prior study has directly compared the efficacy of DFPP versus EFG in gMG patients. Given their fundamentally distinct mechanisms of action—whereas efgartigimod provides selective IgG reduction, DFPP mediates multi‐targeted removal of antibodies, complements, and cytokines—this direct comparison holds considerable clinical and theoretical importance. Based on this, this study conducts a multicenter prospective observational cohort analysis aimed at evaluating the differences in efficacy (via MG‐ADL and QMG scores) and safety between DFPP and EFG. Furthermore, we explored their effects on specific immunological indicators and predictive factors to provide evidence‐based support for individualized treatment strategies in gMG.

## 2 Methods

### 2.1 Ethical Considerations

This study was conducted in strict accordance with the Declaration of Helsinki and was designed as a prospective multicenter observational study with a retrospective data analysis. Starting in August 2019, patients were enrolled and their clinical data were prospectively collected according to a standardized protocol at all participating centers. Informed consent for data usage was obtained from all participants at enrollment. Ethical approval for the final integrated analysis of this prospective database was obtained from the Ethics Committee (No. KS2025‐022) in 2025.

### 2.2 Study Design

This prospective, multicenter, observational cohort study was designed to compare the efficacy and safety of DFPP and EFG in patients with gMG. The patients were recruited from Renji Hospital, School of Medicine, Shanghai Jiao Tong University; Punan Branch of Renji Hospital, Shanghai Jiaotong University School of Medicine (Punan Hospital in Pudong New District, Shanghai); and Anhui No. 2 Provincial People's Hospital. Patient enrollment occurred from August 2019 to March 2025. Data collection, including baseline and follow‐up assessments, continued until June 2025 to monitor treatment efficacy and adverse events.

### 2.3 Study Participants

#### 2.3.1 Inclusion Criteria

(1) Age ≥ 18 years; (2) Diagnosis of MG confirmed by internationally accepted criteria [10], supported by positive serology (AChR‐Ab or MuSK‐Ab) and/or neurophysiological studies (repetitive nerve stimulation or single‐fiber electromyography); (3) Acute exacerbation of gMG, defined as an increase of ≥ 3 points in the MG‐ADL score sustained for at least 48 h compared to a preceding stable baseline, and scheduled to receive either DFPP or EFG treatment; (4) Stable doses of concomitant oral corticosteroids or non‐steroidal immunosuppressive therapies (NSISTs) for ≥ 4 weeks prior to baseline (T0) and maintained through T2, unless clinical worsening required intervention; (5) Complete clinical data and provision of written informed consent.

#### 2.3.2 Exclusion Criteria

(1) Comorbid severe psychiatric or cognitive disorders; (2) Significant dysfunction of the heart, lungs, liver, or kidneys; (3) Malignancy other than thymoma; (4) Pregnancy or lactation; (5) Severe concurrent autoimmune diseases requiring ongoing treatment with other biological agents; (6) Treatment with DFPP, EFG, IVIg, or intravenous methylprednisolone (IVMP) within 1 month prior to enrollment; (7) Incomplete baseline data required for diagnosis or efficacy assessment; (8) Anticipated poor adherence or high risk of loss to follow‐up.

### 2.4 Treatment Allocation

Patients were assigned to either the DFPP or EFG group based on comprehensive clinical considerations, including disease severity, prior treatment response, comorbidities (e.g., contraindications to corticosteroids), patient preference, and healthcare resource availability. Specifically, the choice was guided by (i) the acuity and severity of symptoms (e.g., respiratory involvement favoring rapid intervention), (ii) individual contraindications (e.g., infection risk for DFPP), (iii) informed patient/family preference after detailed consultation, and (iv) practical considerations such as treatment accessibility, cost, and hospital logistics. This allocation reflects real‐world clinical decision‐making rather than randomization.

### 2.5 Treatment Protocols

#### 2.5.1 DFPP Group

Vascular access was established via a central venous catheter. Procedures were performed using the Asahi Kasei Plasauto Σ or Kawasumi KM‐9000 apheresis systems. The exchange volume per session was 1.3–1.5 times the estimated plasma volume (calculated using the Nadler formula), with anticoagulation managed using unfractionated heparin or nafamostat mesylate. Treatments were administered every other day, with a median of 4 sessions (range, 3–6) per cycle. Completion of 3–5 sessions was considered a standard course.

#### 2.5.2 EFG Group

Patients received intravenous EFG at a dose of 10 mg/kg. The planned regimen was once‐weekly infusions for 4 consecutive weeks. The actual number of infusions administered ranged from 1 to 4, with completion of all 4 infusions defined as a standard course.

### 2.6 Outcomes and Endpoints

#### 2.6.1 Primary Efficacy Endpoints

The changes in MG‐ADL and QMG scores were assessed from baseline (T0) to the end of the first treatment cycle (T1) and at the one‐month follow‐up (T2) (Figure S1).

Secondary Endpoints included: (1) The proportion of patients achieving Clinical Meaningful Improvement (CMI), defined as a reduction of ≥ 2 points in MG‐ADL or ≥ 3 points in QMG score sustained for ≥ 4 weeks; (2) The proportion of patients achieving Deep Improvement, defined as either a reduction of ≥ 5 points in the MG‐ADL score or a reduction of ≥ 9 points in the QMG score; (3) Changes in immunological parameters (IgG, IgA, IgM, complements C3, C4, CH50); (4) The incidence of adverse events (AEs).

All clinical scores and blood samples were collected at T0, T1, and T2 (Figure S1). Recorded AEs included type, time of onset, severity (graded by Common Terminology Criteria for Adverse Events, version 5.0, CTCAE V5.0), and outcome.

### 2.7 Laboratory Assays and Sample Collection

Peripheral venous blood samples were collected at baseline (T0), after the first treatment cycle (T1), and at the one‐month follow‐up (T2). Serum was isolated using serum separator tubes. Serum levels of immunoglobulins (IgG, IgA, IgM) and complement components (C3, C4, CH50) were quantified using scatter turbidimetry, while serum cytokines (e.g., IL‐2, IL‐6, IL‐10, TNF‐α) were measured using flow cytometry. For lymphocyte subset analysis, blood was drawn into EDTA anticoagulant tubes to isolate peripheral blood mononuclear cells (PBMCs), which were then characterized by flow cytometry.

### 2.8 Statistical Analysis

To minimize selection bias and confounders, we performed a 1:1 propensity score matching (PSM) analysis. The propensity score was calculated using a logistic regression model based on age, gender, and baseline disease severity (MG‐ADL and QMG scores). We used a caliper width of 0.2 standard deviations of the logit of the propensity score. Covariate balance was assessed using the standardized mean difference (SMD), with an SMD < 0.2 considered indicative of good balance.

Following PSM, a generalized linear mixed model (GLMM) was employed to analyze the primary endpoints (QMG and MG‐ADL scores). The model specification included treatment group, time point, and the time‐by‐group interaction term as fixed effects, while incorporating a subject‐level random intercept to account for within‐subject correlations introduced by repeated measurements. Within‐group comparisons across time points (T0, T1, T2) were performed using paired *t*‐tests or Wilcoxon signed‐rank tests, as appropriate. Between‐group comparisons at each time point utilized independent samples *t*‐tests or Mann–Whitney *U* tests. Categorical variables were compared using the Chi‐square or Fisher's exact test. Additionally, univariate and multivariate logistic regression analyses were conducted to identify predictors of CMI, adjusting for covariates such as age, sex, and baseline Myasthenia Gravis Foundation of America (MGFA) classification. All tests were two‐sided, with a significance level of *p* < 0.05. No adjustment for multiple comparisons was made, and results were interpreted accordingly.

## 3 Results

### 3.1 Patient Baseline Characteristics

**FIGURE 1:**
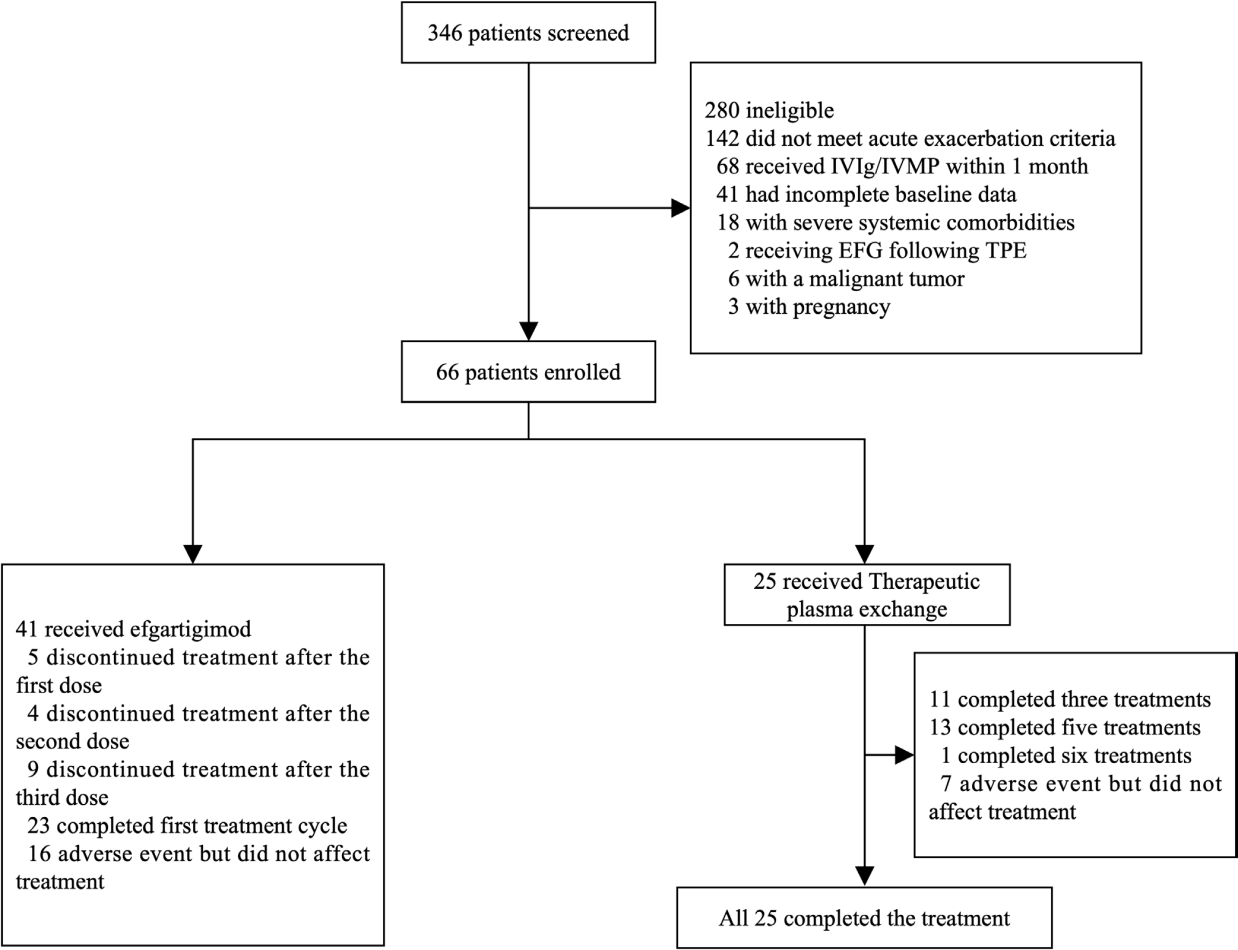
Flowchart of patient screening and enrollment.

A total of 346 hospitalized MG patients were screened between August 2019 and March 2025 at three participating centers. Among the 280 ineligible patients, 251 were excluded for not meeting key inclusion criteria: 142 did not meet the definition of acute exacerbation, 68 had received IVIg or IVMP within the past month, and 41 had incomplete baseline data; presence of significant systemic diseases (*n* = 18), sequential treatment with DFPP followed by EFG (*n* = 2), active malignancy (*n* = 6), or pregnancy (*n* = 3). Consequently, 66 acute exacerbation MG patients were enrolled and received either DFPP or EFG based on clinical circumstances. All 25 patients in the DFPP group completed the planned treatment cycle (3 sessions: *n* = 11; 5 sessions: *n* = 13; 6 sessions: *n* = 1). In the EFG group (*n* = 41), 23 patients (56.1%) completed the full first treatment cycle (4 infusions), while the remaining 18 patients discontinued early (after 1 infusion: *n* = 5; after 2 infusions: *n* = 4; after 3 infusions: *n* = 9). Specifically, Patient decisions were the most frequent reason (*n* = 8), followed by lack of efficacy (*n* = 6). Four patients were lost to follow‐up. Two non‐serious adverse events were reported in each group, none of which led to treatment discontinuation. All 25 patients in the DFPP group and all 41 in the EFG group were included in the full analysis set for both efficacy and safety (Figure 1).

To evaluate the comparative effectiveness of DFPP and EFG in gMG, we first compared the baseline characteristics of the two groups. No significant differences were observed in sex, disease duration, whether it was the first episode, early‐onset MG (EOMG) status, distribution of affected muscle groups, baseline MGFA classification, baseline QMG and MG‐ADL scores, serum antibody profiles (including AChR‐Ab titer), thymoma prevalence, repetitive nerve stimulation (RNS) results, pyridostigmine/corticosteroids dosage during hospitalization, or baseline use of immunosuppressants (all *p* > 0.05), indicating well‐balanced baseline characteristics (Table 1).

**TABLE 1:** Baseline Characteristics of MG Patients Treated with DFPP or EFG.

Variables		DFPP (*n* = 25)	EFG (*n* = 41)	*p*
Age (years)		51.92 ± 14.39	61.63 ± 14.26	0.009^a^
Sex	Male (22)	5 (20.0)	17 (41.5)	0.073
	Female (44)	20 (80.0)	24 (58.5)	
Disease duration (months)		3.00 (1.00, 18.00)	12.00 (2.00, 24.00)	0.090
Initial onset	30/66	15 (60.0)	15 (36.6)	0.064
Early‐onset	23/66	12 (48.0)	11 (26.8)	0.080
Muscles involved				
Limb muscles	28/66	10 (40.0)	18 (43.9)	0.315
Bulbar muscles	18/66	9 (36.0)	9 (22.0)	
Masticatory muscles	9/66	1 (4.0)	8 (19.5)	
Neck muscles	4/66	2 (8.0)	2 (4.9)	
Respiratory muscles	7/66	3 (12.0)	4 (9.8)	
The number of treatments in this cycle		4 (3, 6)	4 (3, 4)	NA
Baseline MGFA	≤ IIa	9 (36.0)	20 (48.8)	0.310
	≥ IIb	16 (64.0)	21 (51.2)	
Baseline MG‐ADL score		8.00 (5.50, 9.50)	6.00 (5.00, 8.00)	0.161
Baseline QMG score		12.00 (6.00, 16.00)	9.00 (6.00, 12.50)	0.137
AChR‐Ab positive	62/66	23 (92.0)	39 (95.1)	0.611
AChR‐Ab titer	50/66	10.92 (2.63, 48.93)	21.86 (5.96, 58.83)	0.478
MuSK‐Ab positive	1/66	1 (4.0)	0 (0.0)	0.161
Titin‐Ab positive	33/66	13 (52.0)	20 (48.8)	0.800
LRP4‐Ab positive	0/66	0 (0.0)	0 (0.0)	—
RyR‐Ab positive	17/66	8 (32.0)	9 (22.0)	0.365
Coexisting other antibodies	8/66	5 (20.0)	3 (7.3)	0.132
Thymoma comorbidity	25/66	13 (52.0)	12 (29.3)	0.065
Thymectomy	12/25	5 (38.5)	7 (58.3)	0.320
RNS	52/66	22 (88.0)	30 (73.2)	0.153
PB during hospitalization (mg/day)	59/66	180.00 (180.00, 180.00)	180.00 (120.00, 210.00)	0.829
Corticosteroid during hospitalization (mg/day)	16/66	23.89 ± 8.58	19.29 ± 7.87	0.289
Baseline DMT	22/66	6 (24.0)	16 (39.0)	0.209

*Note:* Data are presented as mean ± standard deviation, median (interquartile range), or *n* (%), as appropriate.

Abbreviations: DFPP, Double‐Filtration Plasmapheresis; DMT, Disease‐Modifying Therapy; EFG, efgartigimod; MG‐ADL score, Myasthenia Gravis Activities of Daily Living; MGFA, Myasthenia Gravis Foundation of America; PB, Pyridostigmine bromide; QMG score, Quantitative Myasthenia Gravis score; RNS, Repetitive Nerve Stimulation.

^a^ Definitions: Initial onset = the first documented episode of myasthenic symptoms. Early‐onset = disease onset at age ≤ 50 years. Muscles involved = the primary muscle groups affected.

However, initial analysis revealed between‐group differences in several metrics: the age was significantly younger in the DFPP group (51.92 ± 14.39 years) than in the EFG group (61.63 ± 14.26 years, *p* = 0.009; Table 1). Additionally, both the median hospital stay (16.00 [13.50, 19.50] vs. 12.00 [8.00, 16.50] days, *p* = 0.005) and hospitalization costs (5.60 [4.70, 7.25] vs. 5.30 [3.40, 6.50] 10,000 RMB, *p* = 0.046) were higher in the DFPP group. To investigate whether the difference were attributable to the treatment regimens themselves, we repeated the analysis after excluding patients who received non‐standard treatment (DFPP: ≥ 6 sessions or ≤ 2 sessions; EFG: ≤ 3 infusions or ≥ 5 infusions). The results showed that the between‐group differences were no longer statistically significant for either hospital stay (16.50 [13.25, 19.75] days vs. 14.00 [9.00, 18.00] days, *p* = 0.122) or hospitalization costs (5.80 [4.65, 7.38] vs. 5.80 [4.80, 6.80] 10,000 RMB, *p* = 0.840). Detailed data for both the overall cohort and the standardized treatment subgroup are provided in Table S1.

#### 3.1.1 Propensity Score Matching Analysis

To minimize selection bias, a 1:1 PSM was conducted based on age, sex, and baseline severity. This generated 15 pairs of patients (15 in the DFPP group and 15 in the EFG group). In this matched cohort, baseline characteristics were well balanced, with all standardized mean differences (SMDs) significantly reduced to below 0.2. Specifically, the baseline MG‐ADL score (7.3 ± 2.8 vs. 7.2 ± 2.3, SMD = 0.052) and QMG score (10.4 ± 5.3 vs. 9.7 ± 4.1, SMD = 0.140) indicated comparable disease severity between the groups (*p* > 0.05). Detailed baseline comparisons of the matched cohort are presented in Table S2.

### 3.2 Response to the Primary Endpoint

#### 3.2.1 Comparison of Treatment Efficacy

##### 3.2.1.1 Overall (DFPP *n* = 25, EFG *n* = 41)

**FIGURE 2:**
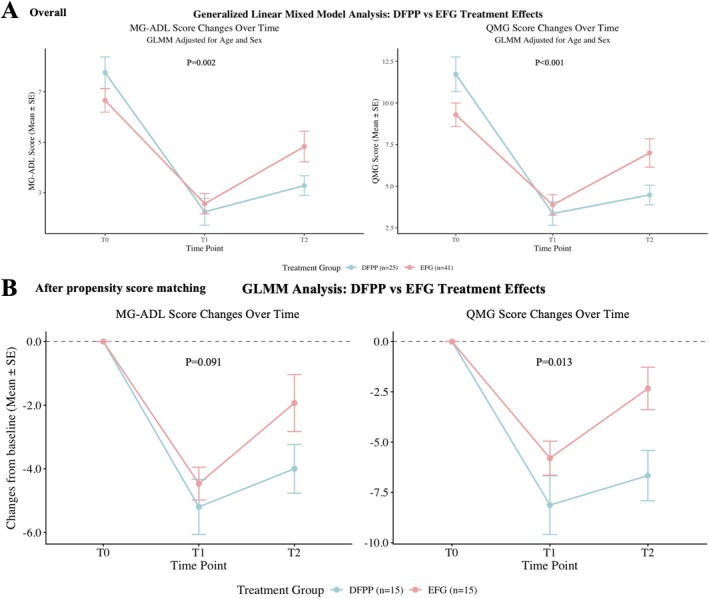
Comparative treatment outcomes between DFPP and EFG groups across different analytical cohorts. Data are presented as mean ± standard error (SE). The plotted *p*‐value represents the time‐by‐group interaction effect. (A) Overall cohort (DFPP *n* = 25, EFG *n* = 41). (B) Propensity score‐matched cohort (DFPP *n* = 18, EFG *n* = 18).

After adjustment for age and sex, the Generalized Linear Mixed Model analysis revealed significant time × group interaction effects for both MG‐ADL score (*p* = 0.002) and QMG score (*p* < 0.001) between the DFPP and EFG treatment groups. Analysis of fixed effects further indicated that the degree of improvement over time was significantly greater in the DFPP group compared to the EFG group (QMG: *β* = −3.50, *p* < 0.001; MG‐ADL: *β* = −1.87, *p* < 0.001). At the T2 time point, the DFPP group demonstrated numerically superior outcomes for both QMG (4.48 ± 0.59 vs. 7.00 ± 0.85; *p* = 0.089) and MG‐ADL scores (3.28 ± 0.39 vs. 4.83 ± 0.61; *p* = 0.089) compared to the EFG group (Figure 2A), though these between‐group differences did not reach statistical significance, potentially due to limited sample size.

##### 3.2.1.2 After Propensity Score Matching (DFPP *n* = 15, EFG *n* = 15)

To account for baseline numerical variations, efficacy was evaluated using a Generalized Linear Mixed Model (GLMM) with a time × treatment interaction term. For QMG scores, a significant time × treatment interaction effect was confirmed (*F* = 3.54, *p* = 0.036). While both groups showed comparable relief at T1 (ΔQMG: 8.13 ± 5.64 vs. 5.80 ± 3.28, *p* = 0.180), the DFPP group demonstrated a significantly greater symptom reduction at the T2 compared to the EFG group (ΔQMG: 6.67 ± 4.85 vs. 2.33 ± 4.10, *p* = 0.014; *β* = −4.33, *p* = 0.010). Regarding MG‐ADL scores, although the overall interaction was not statistically significant (*F* = 1.78, *p* = 0.177), both groups achieved substantial improvement at T1 (5.20 ± 3.34 vs. 4.47 ± 2.00, *p* = 0.473). However, a clear trend toward superior, sustained benefit was observed in the DFPP group at T2 (ΔMG‐ADL: 4.00 ± 2.98 vs. 1.93 ± 3.47, *p* = 0.091; *β* = −2.07, *p* = 0.068), suggesting more sustained clinical benefits (Figure 2B).

### 3.3 Sensitivity Analysis of Efficacy in Per‐Protocol Population

**FIGURE 3:**
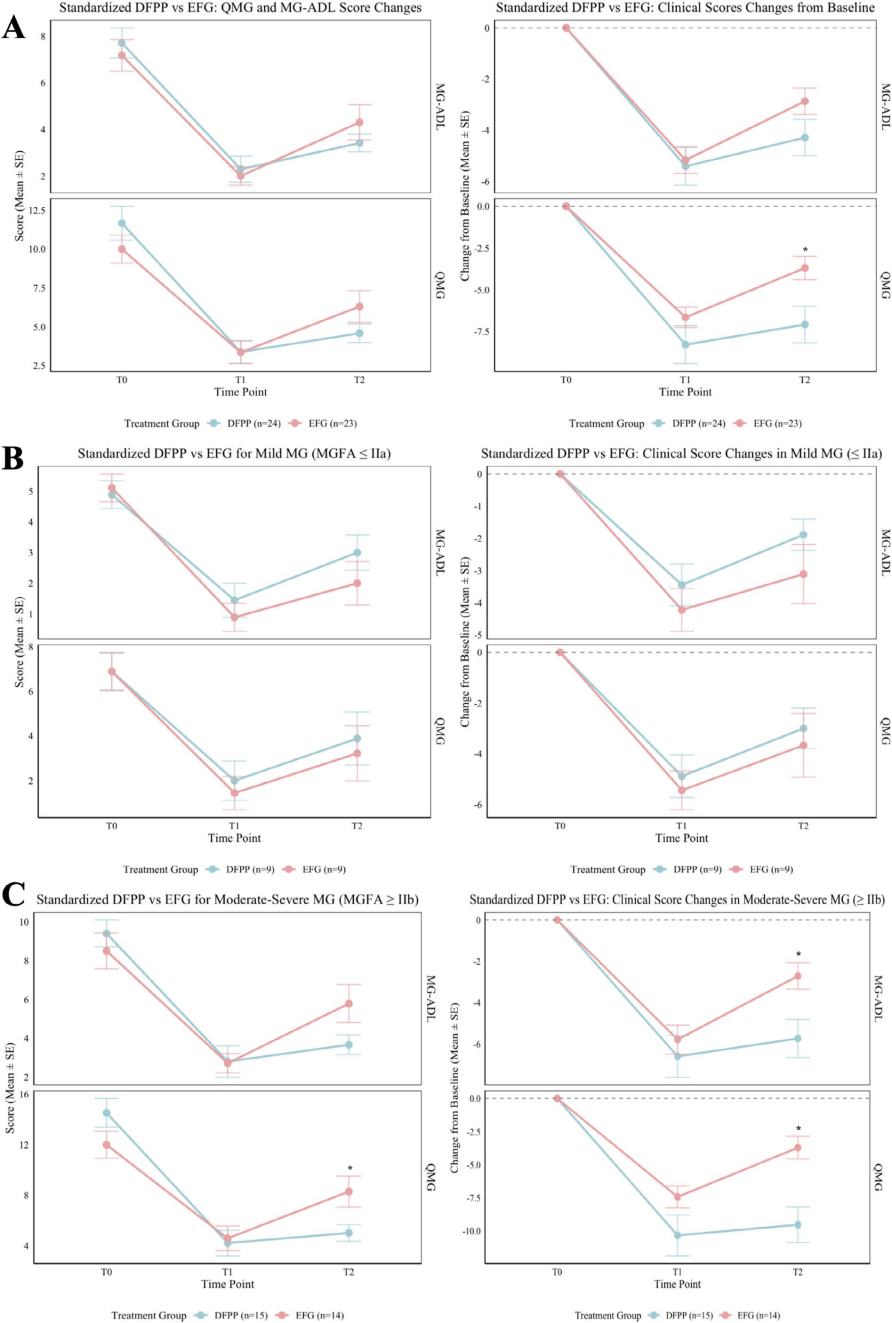
Efficacy Assessment: Sensitivity Analysis and Stratification by Disease Severity. (A) Efficacy comparison of standardized DFPP versus EFG regimens on QMG and MG‐ADL scores. (B, C) Differential treatment efficacy of DFPP versus EFG based on MGFA severity classification. Efficacy was comparable in mild disease but superior with DFPP in moderate‐to‐severe disease at the T2 time point.

Among the 66 enrolled patients, 19 did not complete the per‐protocol treatment (DFPP group: *n* = 1, received > 5 sessions; EFG group: *n* = 18, received ≤ 3 infusions). Consequently, 47 patients constituted the per‐protocol population (DFPP: *n* = 24, 3–5 sessions; EFG: *n* = 23, 4 infusions). Baseline characteristics of the two cohorts were well‐matched (Table S3). Specifically, no significant differences were observed in age (*p* = 0.078), sex (*p* = 0.069), baseline QMG scores (*p* = 0.600), baseline MG‐ADL scores (*p* = 0.465), disease duration (*p* = 0.191), or AChR‐antibody titers (*p* = 0.960). Regarding clinical efficacy, no significant differences were observed at T1 in either the absolute scores or the change from baseline (ΔT1). However, at T2, the improvement in QMG from baseline (ΔT2) was significantly greater in the DFPP group (*p* = 0.013), and a similar trend favoring DFPP was observed for MG‐ADL improvement (Figure 3A).

### 3.4 Stratified Analysis by Disease Severity

In the mild disease subgroup (MGFA class ≤ IIa, *n* = 18), no significant differences were observed between the DFPP (*n* = 9) and EFG (*n* = 9) groups in either the absolute QMG and MG‐ADL scores or the magnitude of change (Δ) from baseline at T0, T1, or T2, indicating comparable efficacy (Figure 3B).

In the moderate‐to‐severe subgroup (MGFA class ≥ IIb, *n* = 29), baseline scores were balanced between the DFPP (*n* = 15) and EFG (*n* = 14) groups. At T2, the DFPP group demonstrated significantly greater improvement than the EFG group in both ΔQMG (*p* = 0.001) and ΔMG‐ADL (*p* = 0.013). Concurrently, the absolute QMG score at T2 was significantly lower in the DFPP group (*p* = 0.030), with a trend toward a lower MG‐ADL score (*p* = 0.069) (Figure 3C).

### 3.5 Impact of Treatment Frequency/Dosage on Efficacy

**FIGURE 4:**
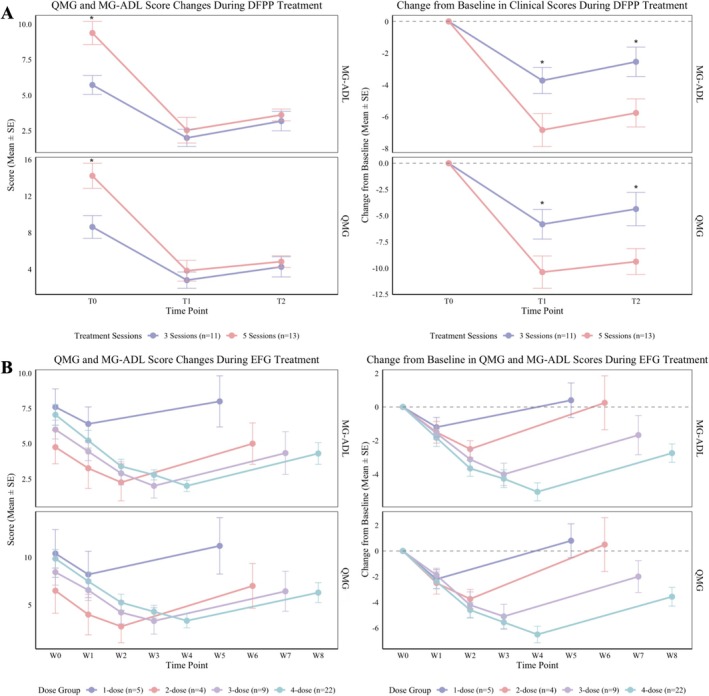
Impact of Treatment Frequency and Dosage on Clinical Efficacy. (A) Comparison of 3 versus 5 sessions of DFPP on QMG and MG‐ADL score improvements. (B) Efficacy of different EFG infusion counts (1–4) over the treatment cycle and follow‐up period.

Within the DFPP group (*n* = 24), the 5‐session regimen (*n* = 13) resulted in significantly greater improvements in both QMG and MG‐ADL scores at T1 and T2 compared to the 3‐session regimen (*n* = 11) (ΔT1 and ΔT2, all *p* < 0.05), despite differences in baseline scores between the two subgroups (Figure 4A).

In the EFG group (*n* = 23), baseline scores were comparable across dosage subgroups (1–4 infusions). The 4‐infusion regimen yielded the greatest improvement at the end of the treatment cycle (Week 4) (ΔQMG: −6.65 ± 0.608, *p* = 0.009; ΔMG‐ADL: −5.17 ± 0.517, *p* = 0.004) and demonstrated better sustained efficacy during the follow‐up period (Weeks 5–8) (Figure 4B).

### 3.6 Response to the Secondary Endpoint

#### 3.6.1 Rates of CMI

**FIGURE 5:**
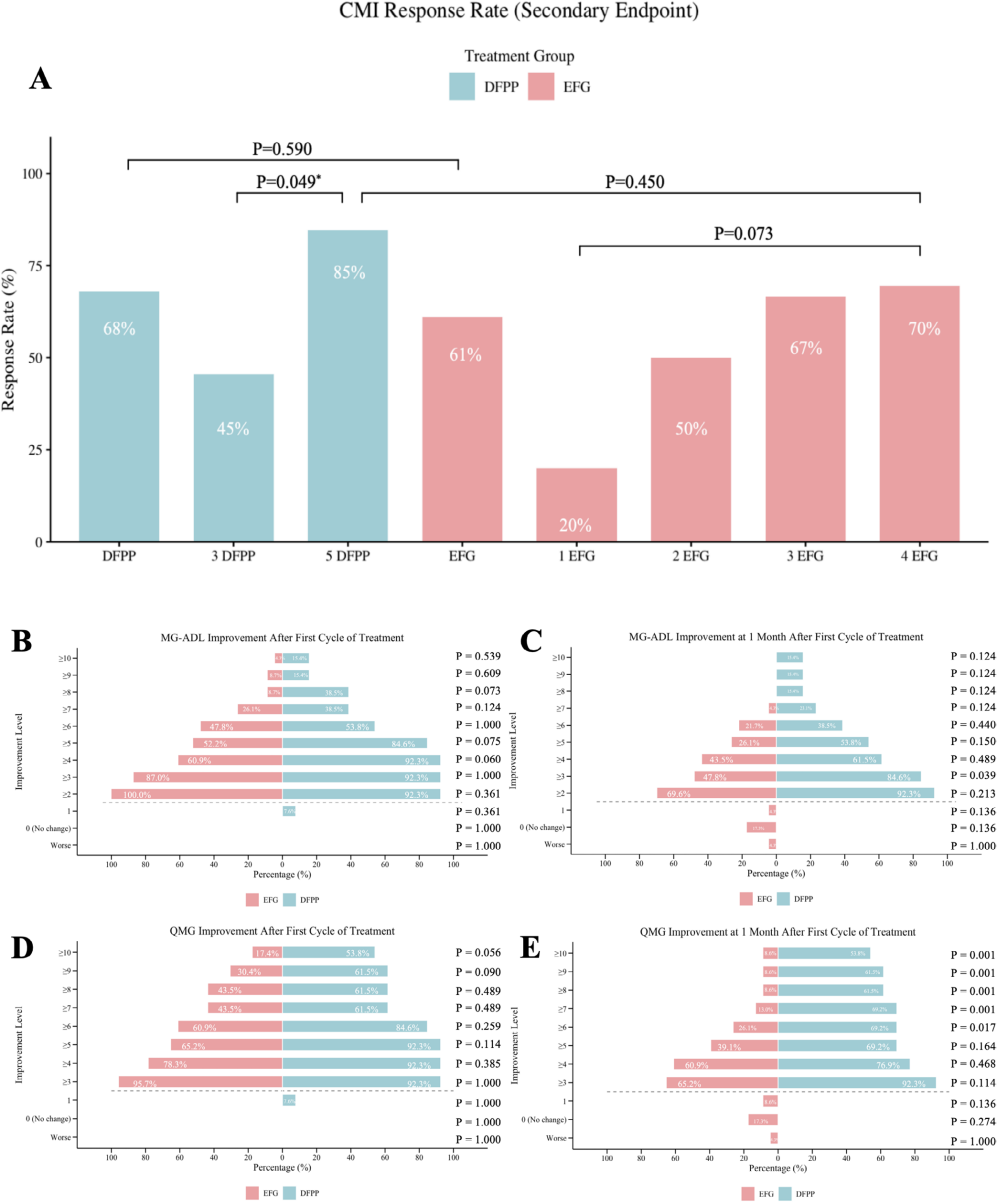
Outcomes of Secondary Endpoints. (A) Rates of Clinical Meaningful Improvement (CMI) by treatment regimen and dose. (B, C) MG‐ADL deep improvement at T1 and T2 timepoints. (D, E) QMG deep improvement at T1 and T2 timepoints.

The overall CMI rates were 68% (17/25) in the DFPP group and 61% (25/41) in the EFG group (*p* = 0.582; Figure 5A). After matching, it demonstrated a comparable overall CMI rate between the DFPP and EFG groups (60.0% [9/15] vs. 66.6% [10/15], *p* = 0.705). Stratified analysis revealed a significantly higher CMI rate in patients who completed 5 sessions of DFPP (85%, 11/13) compared to those who completed 3 sessions (45%, 5/11; *p* = 0.049). The EFG group showed a dose‐dependent increase from 20% (1 infusion) to 70% (4 infusions). Between‐group comparison indicated that the CMI rate was higher in patients completing the 5‐session DFPP regimen (85%) than in those completing the 4‐infusion EFG regimen (70%) (*p* = 0.413; Figure 5A).

Univariate analysis was performed on the standardized treatment cohorts (DFPP, *n* = 24; EFG, *n* = 23), with no clinical factors found to be significantly associated with treatment response in either group (Figure S2).

#### 3.6.2 Proportion of Patients Achieving Deep Improvement

To evaluate the depth of therapeutic response, we compared patients who completed the standardized regimens (5‐session DFPP, *n* = 13; 4‐dose EFG, *n* = 23). At treatment completion (T1), both groups achieved high and comparable response rates for Clinical Meaningful Improvement (CMI): MG‐ADL reduction ≥ 2 (92.3%, 12/13 vs. 100.0%, 23/23; *p* = 0.361) and QMG reduction ≥ 3 (92.3%, 12/13 vs. 95.7%, 22/23; *p* = 1.000). However, the advantage of DFPP became pronounced at the one‐month follow‐up (T2) as the improvement thresholds increased. Regarding the predefined Deep Improvement criteria, the responder rate for QMG reduction ≥ 9 was significantly higher in the DFPP group than in the EFG group (61.5%, 8/13 vs. 8.6%, 2/23; *p* = 0.001). While the proportion of patients achieving an MG‐ADL reduction ≥ 5 was also higher in the DFPP group, the difference did not reach statistical significance (53.8%% vs. 26.1%; *p* = 0.150). Notably, extreme improvement (QMG reduction ≥ 10) remained significantly more frequent in the DFPP group (53.8% vs. 8.6%, *p* = 0.005). These findings, detailed in Table S4, suggest that DFPP facilitates a deeper clinical response in gMG, particularly in objective neurological strength (Figure 5B–E).

### 3.7 Changes in Immunological Parameters

**FIGURE 6:**
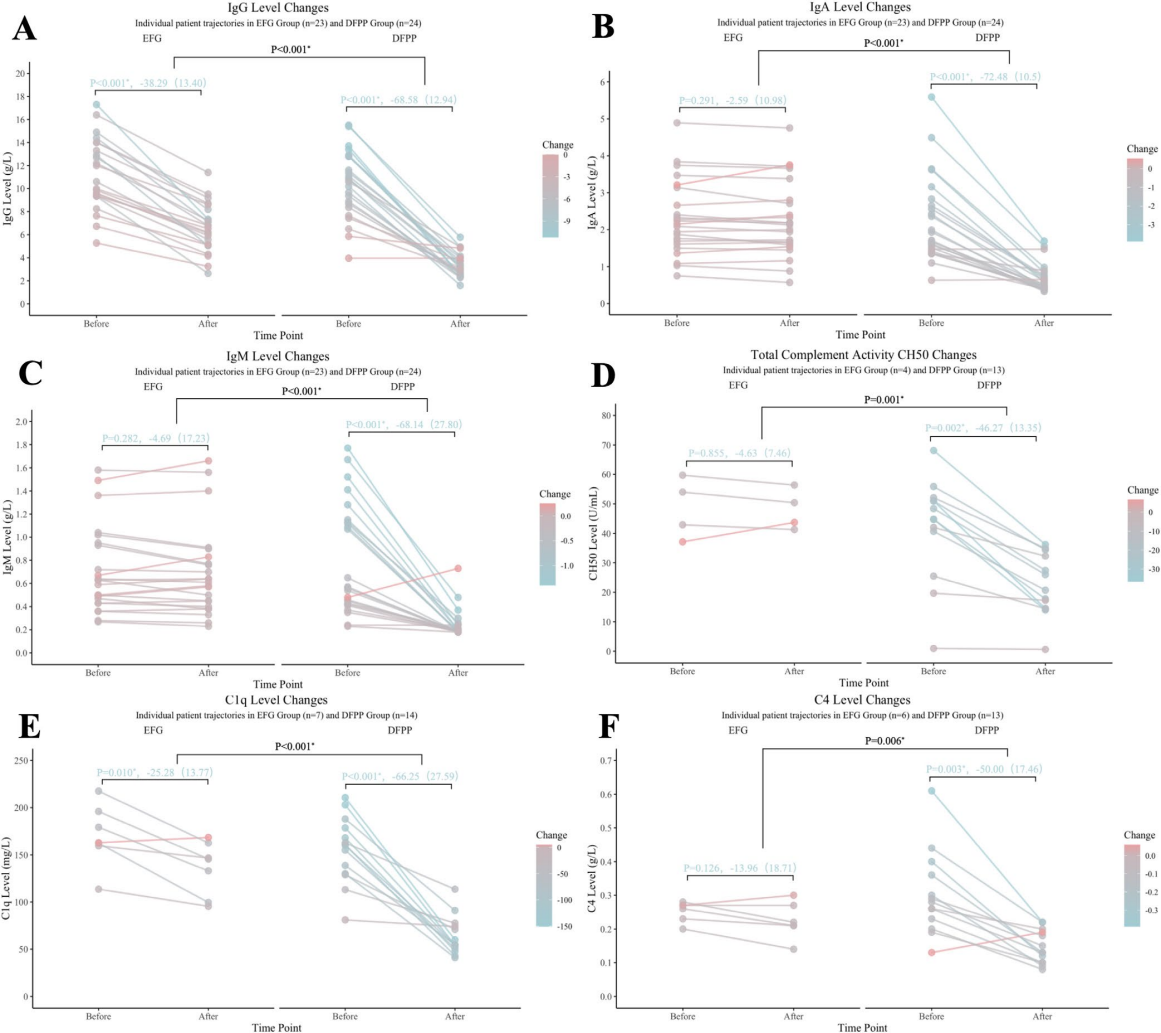
Differential effects of DFPP and EFG on immunological parameters. (A–C) Changes in immunoglobulin levels (IgG, IgA, IgM). (D–F) Changes in complement components (CH50, C1q, C4).

DFPP induced a broad‐spectrum reduction in immunoglobulin levels (IgG, IgA, IgM decreased by 68.1%–72.5%, all *p* < 0.001), whereas EFG selectively reduced IgG (−38.3%, *p* < 0.001) without significant effects on IgA or IgM. The reduction in IgA and IgM was significantly greater with DFPP than with EFG (both *p* < 0.001; Figure 6A–C).

DFPP significantly suppressed the complement system, as evidenced by reductions of 46.3% to 66.3% in 50% complement hemolysis (CH50), C1q, and C4 levels (all *p* < 0.05), while EFG only mildly inhibited C1q (−25.3%, *p* = 0.010). The suppressive effect on complement was significantly stronger with DFPP (all *p* < 0.05; Figure 6D–F).

No significant differences were observed in other lymphocyte subsets or cytokines (all *p* > 0.05; Table S5).

### 3.8 Safety

Both interventions demonstrated a favorable safety profile during the treatment period. The overall incidence of AEs was 28.0% (7/25) in the DFPP group and 39.0% (16/41) in the EFG group (*p* = 0.431), with the vast majority being mild to moderate in severity.

In the DFPP group, the most common AE was catheter‐related thrombosis (12.0%, *n* = 3; Grade 2), followed by systemic infection (8.0%, *n* = 2; Grade 2), hepatic dysfunction (4.0%, *n* = 1; Grade 1), and thrombocytopenia (4.0%, *n* = 1; Grade 1). In the EFG group, headache was the most frequent AE (29.3%, *n* = 12; Grade 1). Other reported AEs included upper respiratory tract infection (4.9%, *n* = 2; Grade 2), mild rash (2.4%, *n* = 1; Grade 1), and herpes zoster infection (2.4%, *n* = 1; Grade 2).

The incidence of infectious AEs was 8.0% (2/25) and 9.7% (4/41) in the DFPP and EFG groups, respectively. No Grade 3 or higher AEs, serious adverse events, or treatment discontinuations due to AEs were reported in either group (Table S6).

## 4 Discussion

This study provides the first direct comparison of DFPP and EFG in gMG, demonstrating comparable efficacy in mild disease but superior and more sustained improvement with DFPP in moderate‐to‐severe cases. This difference in efficacy is likely attributable to the distinct mechanisms of action of the two therapies [11]: DFPP mediates multi‐targeted removal of pathogenic IgG, as well as other immunoglobulins (IgA, IgM), cytokines (IL‐6, IL‐12), and complement components (C1q, C4, CH50), achieving multi‐targeted intervention in the immune network [12]; EFG, in contrast, selectively accelerates IgG degradation by blocking the neonatal Fc receptor (FcRn), achieving targeted immunomodulation with a favorable safety profile [7, 8]. In patients with moderate‐to‐severe gMG, complement‐mediated damage at the neuromuscular junction and polyclonal B‐cell activation play prominent roles [13]; therefore, the broader immune clearance achieved by DFPP may offer unique therapeutic advantages.

Treatment intensity is a key factor influencing efficacy. Among patients receiving DFPP, those who completed five sessions showed significantly greater improvement than those who completed only three sessions, consistent with pharmacokinetic models indicating that 4–5 sessions are required to achieve > 80% reduction in IgG levels [14], suggesting that in patients with high disease burden, an inadequate course of treatment may fail to durably interrupt the immunopathological process. More importantly, the 5‐session DFPP regimen demonstrated superior clinical improvement compared with the 4‐infusion EFG regimen, particularly in patients with moderate‐to‐severe disease. This difference corroborates the mechanistic landscape described above: in the clinical setting of moderate‐to‐severe gMG, where complement‐mediated damage and non‐IgG factors play important roles [13], the broader immune clearance capacity of DFPP translates into tangible clinical advantages. The dynamic modulation of the immune environment by DFPP extends beyond acute clearance. We observed that suppression of complement activity (CH50, C1q, and C4) directly correlated with attenuation of postsynaptic membrane damage [15]. Interestingly, the upward trends in lymphocyte subsets and IL‐2 levels post‐treatment suggest a dynamic immune homeostatic reconstitution process [16, 17, 18, 19]. Regarding the symptomatic fluctuations noted at the one‐month follow‐up (T2), it is important to emphasize that clinical scores remained significantly improved compared to baseline. From a pathophysiological perspective, such transient fluctuations are expected, as DFPP rapidly clears circulating pathogenic antibodies but does not suppress de novo autoantibody production. This rebound phenomenon underscores the role of DFPP as a potent induction therapy: it creates a critical therapeutic window before maintenance immunosuppressive agents achieve full effect, and timely bridging to such agents can consolidate early clinical gains into sustained immune reconstitution [20].

Beyond immunological efficacy, the clinical utility of these therapies is shaped by their economic and safety profiles. Notably, despite being an invasive procedure, DFPP did not significantly prolong hospital stay or increase total costs compared with EFG when standardized treatment regimens were applied, suggesting that it is a cost‐effective option for patients with severe disease. EFG, on the other hand, offers distinct advantages in ease of administration, outpatient suitability, and tolerability, making it particularly suitable for patients with mild disease or those with concerns about invasive procedures. In terms of efficacy depth, the proportion of patients achieving deep improvement was significantly higher in the DFPP group. Importantly, this “deep improvement” carries critical clinical implications for the prevention of myasthenic crisis. In patients experiencing acute exacerbations, achieving rapid and profound symptom relief is essential to forestalling respiratory failure. The more pronounced reduction in QMG scores suggests that for patients on the verge of crisis, the broader immune clearance of DFPP provides greater clinical resilience, effectively bridging the therapeutic gap until maintenance therapies take effect. From a safety perspective, the incidence of adverse events was comparable between the DFPP and EFG groups, with no serious adverse events reported in either group, confirming the favorable safety profile of both therapies in real‐world settings. Taken together, these findings support the implementation of a severity‐stratified treatment strategy: EFG may be prioritized in mild cases, while DFPP may offer deeper and more sustained clinical benefit in moderate‐to‐severe disease.

This study has several limitations. First, treatment allocation was not randomized but based on comprehensive clinical judgment and patient preference; although propensity score matching was applied, selection bias may still exist. Second, the sample size was limited, particularly in subgroup analyses, resulting in insufficient statistical power and a risk of type II error. Third, a considerable proportion of patients in the EFG group did not complete the full four‐infusion course, often due to patient preference or early perceived insufficient response, which is a common real‐world observation and may introduce attrition bias. Future randomized controlled trials with larger sample sizes and longer follow‐up are needed to validate these findings and optimize patient selection strategies. Nevertheless, this study provides the first real‐world direct comparison between DFPP and EFG, offering evidence‐based insights for individualized treatment of gMG.

## 5 Conclusion

In patients with mild gMG, DFPP and EFG demonstrate comparable therapeutic efficacy, whereas DFPP shows superior and more sustained symptomatic improvement in moderate‐to‐severe cases. EFG offers distinct advantages in greater procedural convenience. Based on these findings, we recommend implementing severity‐stratified treatment strategies in clinical practice to achieve personalized therapeutic goals.

## Funding

This study is supported by National Natural Science Foundation of China (No. 82301527, KW); The Young Medical Talents Training Program of Shanghai Pudong New Area Health Commission (No. PWRq2025‐23, KW); Interdisciplinary Program of Shanghai Jiao Tong University (No. YG2023LC04, YG); The Municipal Commission of Health and Family Planning Foundation of Shanghai Pudong New Area (No. PW2022E‐01, YG); New Quality Clinical Specialties of High‐end Medical Disciplinary Construction in Pudong New Area (No. 2024‐PWXZ‐16, YG). The funders were not involved in study conception, data acquisition and interpretation, manuscript preparation, or the decision to submit the article for publication.

## Conflicts of Interest

The authors declare no conflicts of interest.

## Supporting information


**Table S1:** Comparison of Hospitalization Duration and Costs Between DFPP and EFG Groups in the Overall Cohort and Standardized Treatment Subgroup. This table summarizes the length of hospital stay and total hospitalization costs for the entire study population and a specific subgroup that received standardized treatment (3–5 sessions for DFPP or 4 infusions for EFG). It highlights the economic and efficiency differences between the two treatment modalities.
**Table S2:** Baseline Characteristics Before and After Propensity Score Matching. This table presents the clinical and demographic characteristics of patients in the DFPP and EFG groups both before and after applying propensity score matching (PSM). It demonstrates the achievement of covariate balance (SMD < 0.2) to ensure a fair comparison between the two cohorts.
**Table S3:** Baseline Characteristics of the Per‐Protocol Population. This table details the baseline parameters (age, disease duration, MG‐ADL, QMG, and antibody titers) for the subset of patients who strictly adhered to the pre‐defined treatment protocols, serving as the basis for the sensitivity analysis.
**Table S4:** Comparison of Efficacy Between DFPP and Efgartigimod Subgroups Based on Deep Improvement Thresholds. This table compares the proportion of patients reaching “Deep Improvement” (defined as a ≥ 5‐point reduction in MG‐ADL or a ≥ 9‐point reduction in QMG) between the two groups at T1 and T2 time points within the per‐protocol population.
**Table S5:** Longitudinal Changes in Immunological Parameters Following DFPP or EFG Treatment. This table tracks the percentage changes in various immune markers, including T‐cell subsets (CD4+, CD8+, Treg), NK cells, and cytokines (IL‐2, IL‐6, etc.), providing mechanistic insights into how each treatment modulates the immune system.
**Table S6:** Summary of Adverse Events in All Patients. This table provides a comprehensive overview of safety outcomes, listing all treatment‐emergent adverse events (TEAEs) observed in both the DFPP and EFG groups, categorized by type and frequency.
**Figure S1:** Treatment protocol and sample collection timeline for the DFPP and EFG groups. (A) T0 (Baseline): Blood samples were collected before the first DFPP treatment, with simultaneous QMG and MG‐ADL assessments. T1 (Short‐term): At 7 (±3) days following the completion of the first DFPP treatment cycle, blood samples were collected again, and QMG and MG‐ADL assessments were conducted. T2 (Sustained effect): At 4 weeks after the completion of T1 treatment, QMG and MG‐ADL assessments were performed, and blood samples were collected when feasible. All clinical assessments (QMG and MG‐ADL) were completed in a standardized manner at the designated time points. Blood samples were primarily collected during hospitalization (T0, T1). At the T2 time point, as some patients were unable to return to the hospital, follow‐ups were conducted via outpatient assessments or telephone interviews (QMG was assessed in person during outpatient visits, while MG‐ADL could be assessed via telephone). The actual blood sample collection rate at this time point was relatively low. (B) T0 (Baseline): Prior to the initial administration of efgartigimod (corresponding to W0). T1 (Short‐term): At 7 (±3) days following completion of the first efgartigimod treatment cycle (corresponding to W1–W4). T2 (Sustained effect): At 4 weeks after completion of the first efgartigimod treatment cycle (corresponding to W5–W8). At each of the above time points, QMG and MG‐ADL assessments were completed. Blood samples were intended to be collected concurrently at each time point. However, due to practical follow‐up constraints, sample collection was more concentrated among patients who received three or four doses of EFG and were able to return to the hospital for re‐evaluation and continued treatment (i.e., at W3 and W4 time points). Patients who received one or two doses mostly completed clinical assessments via outpatient visits or telephone interviews, resulting in a lower blood sample collection rate.
**Figure S2:** Predictors of Clinical Meaningful Improvement (CMI) identified by logistic regression analysis. (A) Forest plot of univariate logistic regression analysis for CMI in the DFPP groups. (B) Forest plot of univariate logistic regression analysis for CMI in the EFG groups.

## Data Availability

The data that support the findings of this study are available on request from the corresponding author. The data are not publicly available due to privacy or ethical restrictions.
