# Supplementary material for: Double‐Filtration Plasmapheresis Versus Efgartigimod for Generalized Myasthenia Gravis: Severity‐Stratified Benefits in a Prospective Observational Multicenter Study
Source: CNS Neurosci Ther. 2026 Mar 16;32(3):e70838. doi: 10.1002/cns.70838 (PMC13093381; doi:10.1002/cns.70838)
Supplement: Supplementary file 1 — Table S1: Comparison of Hospitalization Duration and Costs Between DFPP and EFG Groups in the Overall Cohort and Standardized Treatment Subgroup. This table summarizes the length of hospital stay and total hospitalization costs for the entire study population and a specific subgroup that received standardized treatment (3–5 sessions for DFPP or 4 infusions for EFG). It highlights the economic and efficiency differences between the two treatment modalities. Table S2: Baseline Characteristics Before and After Propensity Score Matching. This table presents the clinical and demographic characteristics of patients in the DFPP and EFG groups both before and after applying propensity score matching (PSM). It demonstrates the achievement of covariate balance (SMD < 0.2) to ensure a fair comparison between the two cohorts. Table S3: Baseline Characteristics of the Per‐Protocol Population. This table details the baseline parameters (age, disease duration, MG‐ADL, QMG, and antibody titers) for the subset of patients who strictly adhered to the pre‐defined treatment protocols, serving as the basis for the sensitivity analysis. Table S4: Comparison of Efficacy Between DFPP and Efgartigimod Subgroups Based on Deep Improvement Thresholds. This table compares the proportion of patients reaching “Deep Improvement” (defined as a ≥ 5‐point reduction in MG‐ADL or a ≥ 9‐point reduction in QMG) between the two groups at T1 and T2 time points within the per‐protocol population. Table S5: Longitudinal Changes in Immunological Parameters Following DFPP or EFG Treatment. This table tracks the percentage changes in various immune markers, including T‐cell subsets (CD4+, CD8+, Treg), NK cells, and cytokines (IL‐2, IL‐6, etc.), providing mechanistic insights into how each treatment modulates the immune system. Table S6: Summary of Adverse Events in All Patients. This table provides a comprehensive overview of safety outcomes, listing all treatment‐emergent adverse events (TEAEs) observed in bot [file CNS-32-e70838-s001.docx]

### Supplementary Figure 1

Treatment protocol and sample collection timeline for the DFPP and EFG groups.


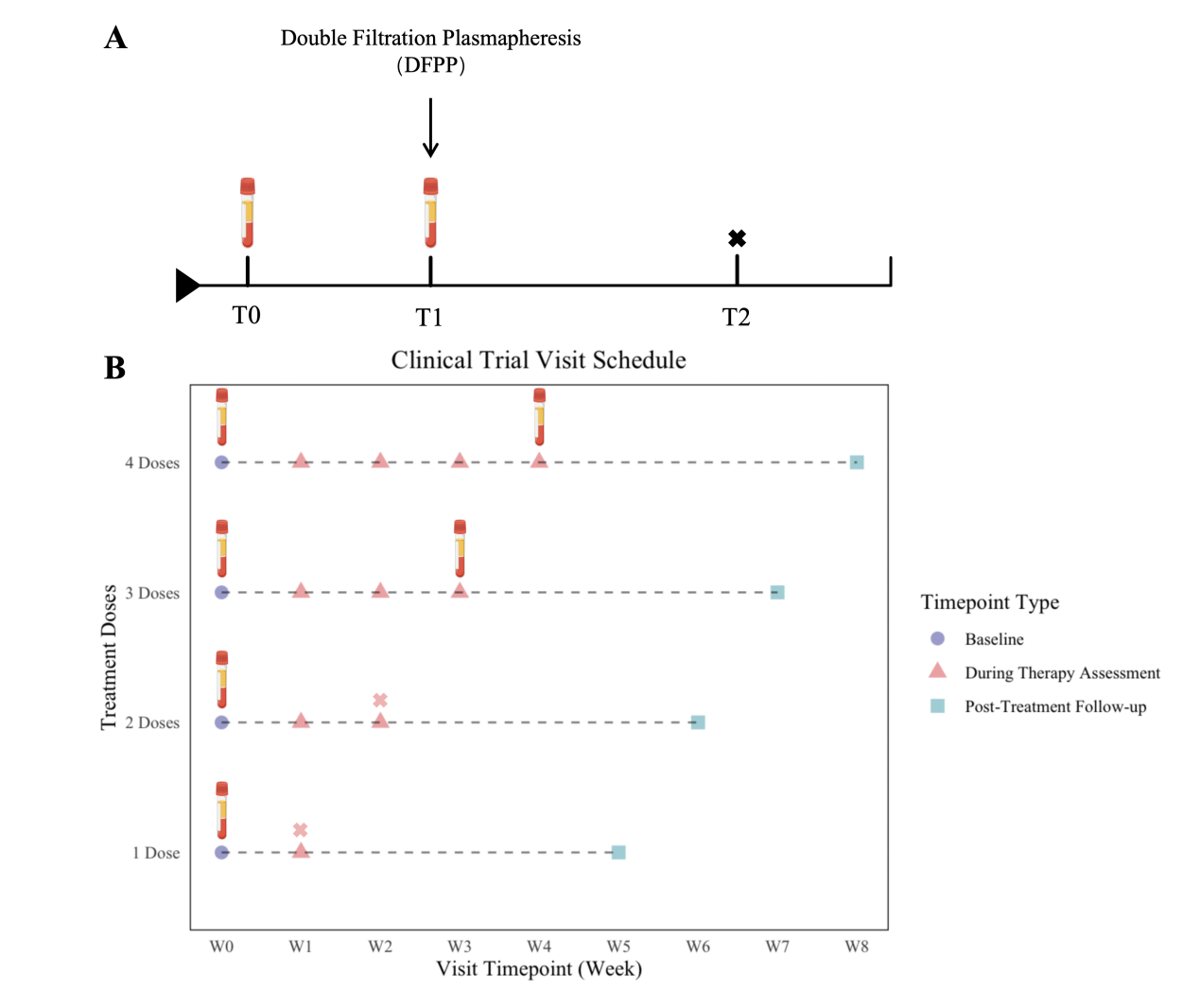


Supplementary Figure 1. Treatment protocol and sample collection timeline for the DFPP and EFG groups.

**A:** T0 (Baseline): Blood samples were collected before the first DFPP treatment, with simultaneous QMG and MG-ADL assessments. T1 (Short-term): At 7 (± 3) days following the completion of the first DFPP treatment cycle, blood samples were collected again, and QMG and MG-ADL assessments were conducted. T2 (Sustained effect): At 4 weeks after the completion of T1 treatment, QMG and MG-ADL assessments were performed, and blood samples were collected when feasible. All clinical assessments (QMG and MG-ADL) were completed in a standardized manner at the designated time points. Blood samples were primarily collected during hospitalization (T0, T1). At the T2 time point, as some patients were unable to return to the hospital, follow-ups were conducted via outpatient assessments or telephone interviews (QMG was assessed in person during outpatient visits, while MG-ADL could be assessed via telephone). The actual blood sample collection rate at this time point was relatively low.

**B:** T0 (Baseline): Prior to the initial administration of efgartigimod (corresponding to W0). T1 (Short-term): At 7 (± 3) days following completion of the first efgartigimod treatment cycle (corresponding to W1-W4). T2 (Sustained effect): At 4 weeks after completion of the first efgartigimod treatment cycle (corresponding to W5-W8). At each of the above time points, QMG and MG-ADL assessments were completed. Blood samples were intended to be collected concurrently at each time point. However, due to practical follow-up constraints, sample collection was more concentrated among patients who received three or four doses of EFG and were able to return to the hospital for re-evaluation and continued treatment (i.e., at W3 and W4 time points). Patients who received one or two doses mostly completed clinical assessments via outpatient visits or telephone interviews, resulting in a lower blood sample collection rate.

**Supplementary Figure 2**


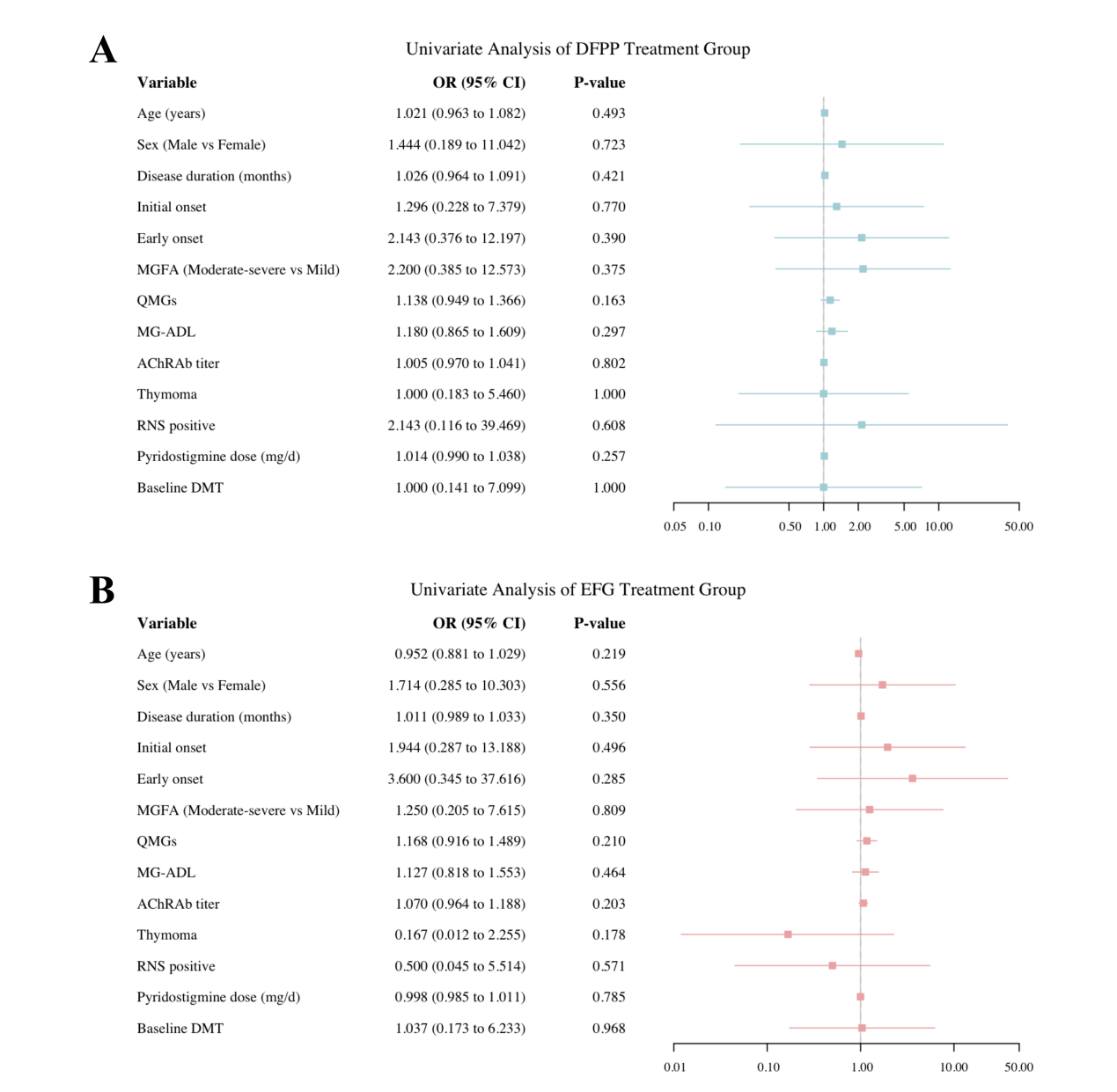
Supplementary Figure 2. Predictors of Clinical Meaningful Improvement (CMI) identified by logistic regression analysis. **(A)** Forest plot of univariate logistic regression analysis for CMI in the DFPP groups. **(B)** Forest plot of univariate logistic regression analysis for CMI in the EFG groups.

**Supplementary Table 1**

Comparison of Hospitalization Duration and Costs Between DFPP and EFG Groups in the Overall Cohort and Standardized Treatment Subgroup

| Variable | Total Patients  (N = 66) | DFPP Group  (n = 25) | EFG Group  (n = 41) | P |
| --- | --- | --- | --- | --- |
| **Overall Cohort** |  |  |  |  |
| Length of hospital stay (days) | 66 | 16.00  (13.50, 19.50) | 12.00  (8.00, 16.50) | 0.005* |
| Hospitalization cost (10,000 RMB) | 66 | 5.60  (4.70, 7.25) | 5.30  (3.40, 6.50) | 0.046* |
| **Standardized Treatment Subgroup** | N = 47 | n = 24 | n = 23 |  |
| Length of hospital stay (days) | 47 | 16.50  (13.25, 19.75) | 14.00  (9.00, 18.00) | 0.122 |
| Hospitalization cost (10,000 RMB) | 47 | 5.80  (4.65, 7.38) | 5.80  (4.80, 6.80) | 0.840 |

Note: Data are presented as Median (Interquartile Range). Standardized treatment is defined as 3–5 sessions for the DFPP group and 4 infusions for the EFG group.

**Supplementary Table 2**

Baseline Characteristics Before and After Propensity Score Matching

| Variable | Unmatched  (EFG n=41, DFPP n=25) | | Matched  (EFG n=15, DFPP n=15) | | | P |
| --- | --- | --- | --- | --- | --- | --- |
|  | DFPP | EFG | DFPP | EFG | SMD |  |
| Age (years) | 51.9±14.4 | 61.6±14.3 | 55.2±14.8 | 54.1±14.3 | 0.078 | 0.834 |
| Female, n (%) | 20（80.0） | 24（58.5） | 10（66.7） | 10（66.7） | <0.001 | 1.000 |
| Baseline MG-ADL | 7.6±3.0 | 6.8±2.8 | 7.3±2.8 | 7.2±2.3 | 0.052 | 0.887 |
| Baseline QMG | 11.3±5.4 | 9.5±4.2 | 10.4±5.3 | 9.7±4.1 | 0.140 | 0.704 |

Notes: Data are presented as mean ± standard deviation (SD) or number (percentage). Abbreviations: DFPP, double-filtration plasmapheresis; EFG, efgartigimod; MG-ADL, Myasthenia Gravis Activities of Daily Living; QMG, Quantitative Myasthenia Gravis; SMD, standardized mean difference. SMD < 0.2 indicates a well-balanced distribution of covariates between the two groups. P-values were calculated using the independent t-test for continuous variables and Fisher's exact test for categorical variables.

**Supplementary Table 3**

Baseline Characteristics of the Per-Protocol Population

| Variables |  | DFPP  （n=24） | EFG  （n=23） | P |
| --- | --- | --- | --- | --- |
| Age (years) |  | 49.67±16.48 | 58.04±15.32 | 0.078 |
| Sex | Male (16) | 5（20.8） | 11（47.8） | 0.069 |
|  | Female (31) | 19（79.2） | 12（52.2） |  |
| Disease duration  (months) |  | 3.00  （1.00，15.00） | 12.00  （1.50，42.00） | 0.191 |
| Baseline MGFA | ≤Ⅱa | 9（37.5） | 9（39.1） | 1.000 |
|  | ≥Ⅱb | 15（62.5） | 14（60.9） |  |
| Baseline MG-ADL score |  | 7.50  （5.75，9.00） | 6.00  （5.00，8.50） | 0.465 |
| Baseline QMG score |  | 10.00  （6.00，16.00） | 10.00  （6.00，12.00） | 0.600 |
| AChR-Ab titer |  | 9.60  （2.71，42.10） | 12.14  （6.22，44.68） | 0.960 |

Note：Data are presented as Mean ± SD, Median [IQR], or n (%). P-values are calculated using Welch's t-test, Mann-Whitney U test, or Fisher's exact test as appropriate. The selected baseline metrics demonstrate comparability between the two cohorts prior to treatment.

**Supplementary Table 4**

Efficacy Comparison of Deep Improvement Thresholds Between DFPP and EFG Subgroups

| Response Threshold | Timepoint | DFPP  (n = 13) | EFG  (n = 23) | P |
| --- | --- | --- | --- | --- |
| **MG-ADL**  **Improvement** |  |  |  |  |
| ≥ 2 points (CMI) | T1 | 12 (92.3%) | 23 (100.0%) | 0.361 |
|  | T2 | 12 (92.3%) | 16 (69.6) | 0.213 |
| ≥ 3 points | T2 | 11 (84.6%) | 11 (47.8%) | 0.039* |
| ≥ 4 points | T1 | 12 (92.3%) | 14 (60.9%) | 0.060 |
| ≥ 5 points  (Deep Improvement) | T1 | 11 (84.6%) | 12 (52.2%) | 0.075 |
|  | T2 | 7 (53.8%) | 6 (26.1%) | 0.150 |
| **QMG**  **Improvement** |  |  |  |  |
| ≥ 3 points (CMI) | T1 | 12 (92.3%) | 22 (95.7%) | 1.000 |
| ≥ 7 points | T2 | 9 (69.2%) | 3 (13.0%) | 0.001* |
| ≥ 8 points | T2 | 8 (61.5%) | 2 (8.6%) | 0.001* |
| ≥ 9 points  (Deep Improvement) | T1 | 10 (76.9%) | 2 (8.6%) | 0.001* |
|  | T2 | 8 (61.5%) | 2 (8.6%) | 0.001* |
| ≥ 10 points | T1 | 7 (53.8%) | 4 (17.4%) | 0.056 |
|  | T2 | 7 (53.8%) | 2 (8.6%) | 0.005* |

Note: Data are presented as n (%). P-values were calculated using Fisher’s exact test. Statistical significance is indicated by * P < 0.05. This analysis includes the per-protocol population who completed the standardized treatment regimens.

Abbreviations: DFPP, double-filtration plasmapheresis (5-session subgroup); EFG, efgartigimod (4-infusion subgroup); MG-ADL, Myasthenia Gravis Activities of Daily Living; QMG, Quantitative Myasthenia Gravis score; CMI, clinical meaningful improvement.

**Supplementary Table 5**

Longitudinal Changes in Immune Parameters Following DFPP or EFG Therapy.

| **Variables** | **N** | **DFPP（P）** | **N** | **EFG（P）** | **P** |
| --- | --- | --- | --- | --- | --- |
| **CD20+（%）** | 16 | 5.06（0.56，22.72）（0.079） | 10 | 2.08（-2.81，8.44）（0.275） | 0.776 |
| **Treg（%）** | 16 | -1.28（-10.10，15.44）（0.860） | 3 | -12.24（-19.04，5.92）（0.784） | 0.359 |
| **B（%）** | 18 | 11.34（1.39，26.56）（0.052） | 12 | 4.43（-1.56，19.35）（0.110） | 0.787 |
| **T（%）** | 18 | 4.21（0.50，8.19）（0.018^*^） | 12 | 4.57（0.85，6.36）（0.064） | 1.000 |
| **Th（%）** | 18 | 7.47（-1.16，17.95）（0.018^*^） | 12 | 4.34（-2.23，11.87）（0.116） | 0.573 |
| **Ts（%）** | 18 | 0.39（-3.42，8.48）（0.298） | 12 | -1.21（-6.27，14.28）（0.733） | 0.787 |
| **CD4+/CD8+** | 18 | 6.94（-4.40，11.65）（0.108） | 12 | 4.13（-4.30，15.43）（0.354） | 0.842 |
| **NK（%）** | 18 | -27.54（-56.33，-2.04）（0.008^*^） | 12 | -19.9（-33.99，10.42）（0.221） | 0.422 |
| **Plasma cells（%）** | 18 | 25.09（-36.82，148.41）（0.246） | 8 | 48.41（-9.4，99.36）（0.151） | 0.644 |
| **C3（g/L）** | 3 | -42.52（-52.07，-11.26）（0.423） | 3 | 7.92（-5.21，8.04）（1.000） | 0.663 |
| **IL-1β（pg/mL）** | 17 | -20.85（-49.43，8.13）（0.222） | 8 | -30.60（-56.82，-15.54）（0.054） | 0.398 |
| **IL-2（pg/mL）** | 17 | 21.72（-12.89，119.05）（0.041^*^） | 8 | -37.28（-65.76，26.46）（0.700） | 0.086 |
| **IL-4（pg/mL）** | 17 | 0.00（-23.50，43.27）（0.299） | 8 | -21.79（-41.79，-3.12）（0.118） | 0.063 |
| **IL-5（pg/mL）** | 17 | -2.57（-43.18，58.25）（0.525） | 8 | 3.86（-30.53，23.13）（0.953） | 0.647 |
| **IL-6（pg/mL）** | 17 | 32.07（-10.13，48.36）（0.074） | 8 | -21.21（-40.13，11.14）（0.913） | 0.140 |
| **IL-8（pg/mL）** | 17 | -5.98（-51.58，9.78）（0.552） | 8 | -18.32（-26.93，7.48）（0.774） | 0.754 |
| **IL-10（pg/mL）** | 17 | 11.27（-6.86，70.03）（0.083） | 8 | -7.16（-28.58，19.08）（0.718） | 0.281 |
| **IL-12p70（pg/mL）** | 17 | 22.30（0.00，69.74）（0.057） | 8 | -5.87（-30.54，24.54）（0.963） | 0.210 |
| **TNF-α（pg/mL）** | 17 | -23.21（-41.91，21.05）（0.423） | 8 | -10.60（-32.41，-3.81）（0.247） | 0.842 |
| **IFN-α（pg/mL）** | 17 | 0.00（-14.53，19.23）（0.798） | 8 | -27.53（-51.38，12.98）（0.366） | 0.256 |

Note：Data are presented as mean percentage change ± standard deviation（SD）percentage. P values (within-group) reflect pre-post treatment changes (paired t-test or Wilcoxon test). P values (between-group) reflect differences in the change between DFPP and EFG (Student's or Welch's t-test, based on variance equality). DFPP= Double Filtration Plasmapheresis. EFG= efgartigimod. CD20+ = CD20+ B lymphocytes. Treg = Regulatory T cells (CD3+CD4+CD25+CD127-). B = B lymphocytes (CD3-CD19+). T = T lymphocytes (CD3+). Th = T helper lymphocytes (CD3+ CD4+). Ts = T helper lymphocytes (CD3+ CD4+). CD4+/CD8+ = Ratio of T helper to T suppressor/cytotoxic cells. NK = Natural killer cell (CD3-CD16+CD56+). C3 = Complement component 3. IL-1β = Interleukin-1 beta. IL-4 = Interleukin-4. IL-5 = Interleukin-5. IL-6 = Interleukin-6. IL-8 = Interleukin-8. IL-10= Interleukin-10. IL-12p70 = Interleukin-12, bioactive heterodimer (composed of p35 and p40 subunits) . TNF-α = Tumor necrosis factor-alpha. IFN-α = Interferon-alpha.

**Supplementary Table 6**

Summary of adverse events in all patients.

| DFPP group（n=25） | | EFG group（n=41） | |
| --- | --- | --- | --- |
| Any adverse event | 7（28.0%） | Any adverse event | 16（39.0%） |
| Any infection | 2（8.0%） | Any infection | 4（9.7%） |
| Most common adverse events |  | Most common adverse events |  |
| Catheter-related throm bosis | 3（12.0%） | Headache | 12（29.3%） |
| systemic infection | 2（8.0%） | Upper respiratory tract infection | 2（4.9%） |
| hepatic dysfunction | 1（4.0%） | Mild rash | 1（2.4%） |
| thrombocytopenia | 1（4.0%） | Herpes zoster infection | 1（2.4%） |

Note：Data are presented as the number of patients with the event (n) and percentage (%). DFPP = Double Filtration Plasmapheresis. EFG = efgartigimod. All adverse events occurring during the treatment period were recorded and are listed herein.
